# Isolation and Characterization of Simple Sequence Repeats (SSR) Markers from the Moss Genus *Orthotrichum* Using a Small Throughput Pyrosequencing Machine

**DOI:** 10.3390/ijms13067586

**Published:** 2012-06-19

**Authors:** Jakub Sawicki, Mirosław Kwaśniewski, Monika Szczecińska, Karolina Chwiałkowska, Monika Milewicz, Vítězslav Plášek

**Affiliations:** 1 Department of Botany and Nature Protection, University of Warmia and Mazury in Olsztyn, Plac Lodzki 1, Olsztyn 10-727, Poland; E-Mails: monika.szczecinska@uwm.edu.pl (M.S.); monika.milewicz@uwm.edu.pl (M.M.); 2 Department of Genetics, University of Silesia, Jagiellonska 28, Katowice 40-032, Poland; E-Mails: kwasniew@us.edu.pl (M.K.); karolina.chwialkowska@us.edu.pl (K.C.); 3 Department of Biology and Ecology, University of Ostrava, Chittussiho 10, Ostrava 71000, Czech Republic; E-Mail: vitezslav.plasek@osu.cz

**Keywords:** SSR markers, *Orthotrichum*, 454 pyrosequencing

## Abstract

Here, we report the results of next-generation sequencing on the GS Junior system to identify a large number of microsatellites from the epiphytic moss *Orthotrichum speciosum*. Using a combination of a total (non-enrichment) genomic library and small-scale 454 pyrosequencing, we determined 5382 contigs whose length ranged from 103 to 5445 bp. In this dataset we identified 92 SSR (simple sequence repeats) motifs in 89 contigs. Forty-six of these had flanking regions suitable for primer design. We tested PCR amplification, reproducibility, and the level of polymorphism of 46 primer pairs for *Orthotrichum speciosum* using 40 individuals from two populations. As a result, the designed primers revealed 35 polymorphic loci with more than two alleles detected. This method is cost- and time-effective in comparison with traditional approaches involving cloning and sequencing.

## 1. Introduction

Due to their high polymorphism and reproducibility, co-dominant SSR (simple sequence repeats) markers are widely used in population genetics and phylogeographic studies [1]. SSR markers are also applied to determine the taxonomic status of species at the early stages of divergence [2].

Despite their numerous advantages, SSR markers can still be problematic to use. The drawback of highly-specific SSR markers is their laborious development. The traditional method of developing SSR markers is both labor-consuming and expensive, and it often generates a small number of polymorphic loci [3]. Methods in which non-specific markers such as AFLP [4,5], ISSR [6] and RAPD [7] are used for enrichment are also commonly employed, which does not exclude the cloning process and clone screening.

Next-generation sequencing has been used recently to isolate SSR markers [8,9]. High-throughput next-generation sequencers support the isolation of several hundred polymorphic loci in a single run [10]. This approach is usually followed in animal studies, but attempts have also been made to apply the above method for the isolation of SSR markers from plants. However, population studies seldom require such a large number of loci, and the cost of a single run on the Roche 454 GS FLX sequencer (Roche 454 Life Sciences, Branford, USA), typically used for this purpose, is relatively high.

We have successfully isolated, at little cost and effort, several dozen SSR markers (sufficient to conduct thorough population studies) with the use of the GS Junior 454 system (Roche 454 Life Sciences, Branford, CT, USA). The total cost of such an analysis ranges from EUR 1000 to 1200 (April 2012).

SSR markers have rarely been developed for bryophytes, since most bryologists use AFLP, ISSR and RAPD markers [11–13]. However, specific markers are increasingly applied due to a growing awareness that research results may be affected by the presence of biological pollutants [14]. To date, conventionally generated SSRs have been developed for a few moss species of the genera *Sphagnum* [6] and *Platyhypnidium* [15]. This paper describes the development of SSR markers for the epiphytic moss species *Orthotrichum speciosum*. The markers will be employed in phylogeographic and ecological studies aimed at evaluating environmental pollution based on the genetic variability of *O. speciosum* populations.

## 2. Results and Discussion

A single sequencing run of *Orthotrichum speciosum* DNA library in the GS Junior pyrosequencing system resulted in 139,886 reads with an average read length of 426 bp. In total, 59,645,460 high-quality base-pairs were obtained. Sequence assembling and mapping to the chloroplast genome of *Syntrichia ruralis* allowed the alignment of 814 reads and their contigs to the reference genome; the N50 contig size (statistical measure of average length of a set of sequences) was 939 bp. The *Orthotrichum speciosum* sequences obtained in the analysis covered the chloroplast genome of *Syntrichia ruralis* in 50.3%, at an average depth of 1.6. The remaining reads were de novo assembled into 5382 contigs with a length of 103 to 5445 bp.

An analysis of the obtained sequences with the use of msatcommander enabled us to determine the location of 92 SSR motifs in 86 contigs. Tri- (49) and di-nucleotide (27) repeats dominated among the discovered microsatellite motifs. Longer repeat motifs included 7 tetra- and 2 hexa-nucleotide ones.

In several cases, primers could not be designed since motifs were located at the edges of contigs. Finally, we used 46 pairs of primers, 35 of which were found to be polymorphic (Table 1). The primers revealed from 3 to 9 alleles per locus, 3.77 alleles on average. The values of the Nei’s genetic diversity coefficient [16] in the test sample ranged from 0.210 to 0.550. Significant LD occurred in the studied populations for only one pair of loci, os8 and os24 (*p* < 0.05).

Nearly half of the tested SSR markers amplified also single bands in closely related species of the subgenus *Gymnoporus*. In *O. affine* and *O. striatum*, 18 and 17 pairs of primers, respectively, amplified single loci (Table 1), which testifies to the close relationship between the species [17]. Cross-amplification was considerably less successful in phylogenetically distant species of the subgenus *Pulchella*, where the primers were effective in 8 (*O. pallens*) and 6 (*O. diaphanum*) cases.

## 3. Experimental Section

### 3.1. Plant Materials

The genus *Orthotrichum* is a widespread moss group, which includes approximately 159 species [18], and is the second largest genera in the family Orthotrichaceae. Taxa belonging to this genus are found throughout the world from the Arctic to the Antarctic, except in deserts and wet tropical forests. Species of the genus *Orthotrichum* grow on trees and rocks to an elevation of *ca.* 5000 m above sea level [19]. The subdivision within this genus has been a matter of a continuing debate since the end of the 19th century. Certain taxa have been alternately included in and excluded from the genus *Orthotrichum* in the attempt to divide it into lower taxonomic units, subgenera and sections. The basis for the classification of the genus *Orthotrichum* in a historical perspective has been described in detail by Lewinsky [19] and Lewinsky-Haapasaari and Hedenäs [20].

*Orthotrichum* species have a wide geographical range and are usually characterized by high genetic diversity, in some cases pointing to the occurrence of cryptic species [17,18]. Several widespread species, including *O. speciosum* of the subgenus *Gymnoporus*, showed a very low level of genetic variation in the analyzed regions. *O. speciosum* is a common representative of the genus, found across the entire Holarctic ecozone. The species is well defined morphologically [19,21] and genetically [17], and it is characterized by a low level of genetic variation in nuclear and chloroplast sequences [17,22], which is why it has been selected as a model species for the present study. The developed markers will also be used in ecological studies, to replace less polymorphic ones. A population of *O. speciosum* from the Czech Republic was used for DNA isolation (Kouty nad Desnou, Hruby Jesenik Mts).

### 3.2. DNA Extraction

Total genomic DNA was extracted from 30 fresh stems. The stems were ground with silica beads in a MiniBead-Beater tissue disruptor for 50 seconds, and were subsequently processed using the DNeasy^®^ Plant Mini Kit (Qiagen, Hilden, Germany) following the manufacturer’s protocol. DNA quantity was estimated with the Qubit fluorometer system (Invitrogen, Carlsbad, NM, USA), using the Quant-IT ds-DNA BR Assay kit (Invitrogen).

### 3.3. DNA Library Preparation and Sequencing

Eight hundred nanograms of DNA was sheared by nebulization, purified with the MinElute PCR Purification Kit (Qiagen), and subsequently processed according to the GS Rapid Library Preparation Kit Method Manual (Roche/454 Life Sciences). The quality of DNA library was assessed by gel electrophoresis in the FlashGel System (Lonza). DNA fragments were clonally amplified using the GS Junior Titanium emPCR Lib-L Kit (Roche/454 Life Sciences). Sequencing was performed using the GS Junior pyrosequencing system according to the Sequencing Method Manual (Roche/454 Life Sciences).

Pyrosequencing data were assembled using GS Reference Mapper software (Roche/454 Life Sciences). A two-step assembly was performed. First, the obtained sequences were assembled using the chloroplast genome data of *Syntrichia ruralis* (GenBank: NC_012052.1) to separate chloroplast reads from nuclear reads. *Syntrichia ruralis* is one of the two moss species with sequenced cpDNA genomes and is closer related to *Orthotrichum* than the *Physcomitriella patens*. The remaining reads were assembled using the GS Newbler *de novo* assembler (Roche/454 Life Sciences).

The obtained contigs were searched for microsatellite motifs using msatcommander with default settings [23]. This program was also used for primer design. To avoid designing primers for any potential SSR locus twice, the contigs containing the same motif were compared in Bioedit 7.0.5 [24].

### 3.4. Genotyping Test

We tested PCR amplification and the level of polymorphism of the designed primer pairs. The sequences used in genotyping test were deposited in GenBank (accession numbers from JX154169 to JX154203). The polymorphism of SSR markers was tested in two *O. speciosum* populations of 20 specimens each, and in the material used in our previous studies [17,22]. The cross-species amplification of SSR loci was tested in both closely related *O. affine* and *O. striatum*, and more phylogenetically distant *O. diaphanum* and *O. pallens* [25,26].

SSR-PCR reactions were performed in 20 μL of a reaction mixture containing 40 ng genomic DNA, 1.0 μM of each primer, 1.5 mM MgCl_2_, 200 μL M dNTP (dATP, dGTP, dCTP, dTTP), 1× PCR buffer, 1 μL BSA and 1 U Genomic RedTaq polymerase (Sigma, St. Louis, USA). SSR marker reactions were performed under the following thermal conditions: (1) initial denaturation—5 min at a temperature of 94 °C; (2) denaturation—1 min at 94 °C; (3) annealing—1 min at 53 °C; (4) elongation—1 min at 72 °C, final elongation7 min at 72 °C. Stages 2–4 were repeated 34 times. The products of the PCR reaction were separated on the QIAxcel capillary electrophoresis system, which is a cost-effective system suitable for SSR marker electrophoresis [27]. Electrophoresis was performed using the Qiaxcel High Resolution Kit with the alignment marker 15–500 bp (Qiagen) and the DNA size marker 25–500 bp (Qiagen). Standard OM700 settings were used as the electrophoresis program.

To check consistency of designed primers, randomly selected 24 amplicons were resequenced using amplification primers. Purified PCR products were sequenced in both directions using the ABI BigDye 1.1 Terminator Cycle Kit (Applied Biosystems, Foster City, USA), and were visualized using an ABI Prism 3130 Automated DNA Sequencer (Applied Biosystems).

An analysis of genetic diversity was performed using PopGen 1.32 software [28]. The linkage disequilibrium (LD) was tested using FSTAT v.2.9.3 [29].

## 4. Conclusions

The genetic resources of epiphytic mosses have declined due to air pollution and excessive tree cutting. The development of SSR markers from *O. speciosum* and related species open new possibilities in studying their genetic variation, phylogeography and populations structure. The SSR loci reported here are the first SSR markers to be designed specifically for species belonging to the Orthorichaceae family, and the third moss species. The method described in this paper allowed us to obtain at least 35 polymorphic loci, at a total cost of approximately EUR 1000–1200, using a fast and easy approach.

## Figures and Tables

**Table 1:** Characteristics of simple sequence repeats (SSR) loci for *Orthotrichum speciosum* (H–Nei’s genetic diversity, *Oa*—*Orthotrichum affine*, *Os*—*O. speciosum*, *Op*—*O. pallens*, *Od*—*O. diaphanum*).

Locus	Motif	Primers	Product Size	Diversity		Cross-Amplification			
	
				Number of Alleles	H	*Oa*	*Os*	*Op*	*Od*
os1	(GTT)_4–7_	F-GCAACTTCCTCCAACGACC R CAGATTGCGGCTGACCAAG	378–387	3	0.405	+	-	-	-
os2	(GT)_6–12_	F-CAAACACGACCGCTTCTCC R-GAGAGCTATCTCCCTCGAAAG	405–417	6	0.540	-	-	-	-
os3	(AGG)_4–8_	F-GTACGTCGTGCCCAAATCG R-CGTCGCATTCCCACAGAAG	354–366	5	0.355	+	+	-	-
os4	(AAT)_4–6_(AT)_7–15_	F-CACTCAAGTGAAGAGTCATGGG R-CGAGCAACGTGGCATGAAC	329–351	9	0.380	-	-	-	-
os5	(AT)_5–12_	F-AGGATTGATTGCCTTTGCGG R-GATCATTCGCATCTGGGCG	229–243	5	0.290	-	-	-	-
os6	(AG)_6–11_	F-GTTGACGAAGCCCTCTTGG R-CTTTGAGACGTGGTAATCTGAAG	411–421	7	0.550	-	-	-	-
os7	(ATT)_4–7_…(AAT)_5–7_	F-TTCAACCATGTGCTAGTTGTATC R-AGGGTCCAAACTCTAAACTGAC	414–425	5	0.285	-	+	-	-
os8	(CTT)_4–8_	F-TTCCCTTCAACCGCCACTC R-CCGAAGGCTGGATAATTGCC	263–275	3	0.230	+	+	+	+
os9	(CGT)_4–7_	F-GGCCATTGAAAGCAGGCTC R-CGGCTACGACATCAATGAAAG	401–410	3	0.280	+	+	-	-
os10	(ACC)_6–10_	F-CCTCGTAGGGTATCTCCGC R-ATCAAGAGTCGGGACGTGG	243–255	4	0.305	-	-	-	-
os11	(GTT)_4–10_	F-GCGTTGTGGAGTAAGGACTG R-CCCATCACCACTATGATGCC	202–220	5	0.410	-	-	-	-
os12	(AAAT)_4–6_	F-AATGTTGGAAACCAGCCCG R-TCCGGATTAGAAGATTTACAGTGG	158–166	3	0.210	-	+	-	-
os13	(AG)_6–10_	F-AGAATTGCTACTACATGAACGTG R-TTGTGTCCCGTCCCTCAAC	192–200	3	0.430	+	+	+	+
os14	(AAC)_6–9_	F-CTCCGAGTCCACTTGGTCG R-GACTGAAGTGCTGGCTTGG	198–210	3	0.250	+	-	-	-
os15	(AAAG)_6–8_	F-TGAAGTATCCAGACCAAGAGC R-ACATTCTGCCCTCAATGTCG	152–160	3	0.220	-	-	-	-
os16	(AAG)_4–7_	F-AAGAAGGCGTCAGCTTCAC R-TAGCTGCCCGCAACTTC	248–257	3	0.290	+	+	-	-
os17	(GAT)_4–7_	F-AGCGAGTTGATGGCGGAG R-TCCTCCAATGCCTTAGTCAAAC	361–370	3	0.340	-	+	-	-
os18	(GTT)_4–6_	F-CATGATGCTGCCCTTGTCC R-GTTAGCTGCATGTCACGGC	307–313	3	0.510	+	+	+	-
os19	(CTT)_4–6_	F-CCCACGCCACTTAGTCTTG R-GGAGAATGACAACCTCAGCC	229–235	3	0.260	+	+	+	+
os20	(ATTT)_6–9_	F-AGTTGTGTCTTCCTTCATCTATACC R-GATGGGCCAAAGTGTCTCG	169–181	3	0.220	-	-	-	-
os21	(CTT)_5–9_	F-AGCGAGTGTACATCCGAGC R-GCCTAAGCCCACTTGGAAAC	193–205	4	0.290	+	+	+	-
os22	(GCT)_4–7_	F-AAATCTACAACTTCGCACGTC R-TGAGATTCATGAGAGGTGTCCG	161–170	3	0.310	-	-	-	-
os23	(AT)_7–12_	F-TTCATTGTCCTAAGATTCCC R-GATGCAANTACGTCTTATAATC	202–212	5	0.490	-	-	-	-
os24	(ATT)_7–11_	F-GTTGAAATCTACTANAAAAGTT R-GCTCNAAATCNCATCTAANCT	181–193	3	0.230	+	-	-	-
os25	(GTT)_4–6_	F-GGAGTCCCTCCAGCAAGTATG R-GCGNCTAGGTCATGTACTNATGG	326–335	3	0.260	-	+	-	-
os26	(GTC)_5–8_	F-ACTTGCTGAAGAACGGTCTGC R-GTAACGTCTTGTCACTGAC	298–307	3	0.290	+	+	-	-
os27	(GT)_6–11_	F-CCTTCATTCCATTTGCCCNTTG R-GTATGTTGCCTCCTCCAATTCATT	201–211	4	0.370	-	-	-	-
os28	(GA)_6–10_	F-TTCTCCATGTTCTCTACTTNGG R-GACGGCCTCTCGGCAAGAGTTTG	210–218	3	0.220	+	+	+	+
os29	(GA)_7–10_	F-CATCAATGATGTAGGATNGAAN R-CTCAATATCTGGATTTCTGGGA	197–203	3	0.280	+	+	+	+
os30	(CA)_11–16_	F-ACACACNCANACACACACNCNC R-TGGATGCGTGTGGGCACCTGT	260–270	4	0.410	-	-	-	-
os31	(GAT)_4–7_	F-CGTTGATTCTATTTGATAGCTAA R-TTGACATGTCTGAGCCCC	241–250	3	0.320	+	+	+	+
os32	(AAAT)_4–6_	F-NCCNANCCATGTCAGAAAAAG R-GCCGCATTATGAAGTTGGA	269–278	3	0.220	-	-	-	-
os33	(ATT)_4–6_	F-CTACAATAAGAGCTCTTTGAA R-ACANTTTGGATCTCAGCCTG	202–208	3	0.260	-	-	-	-
os34	(GAT)_4–6_	F-AGGGCTCTANCTTATAGNTTG R-GAGGTGGACAGTGCAAGTGNAAG	210–216	3	0.230	+	+	-	-
os35	(GGA)_4–7_	F-CCCGAGTCCACTTGGNANCC R-GCTAAGCCCAGTTAGAAGCTC	171–180	3	0.345	+	-	-	-
